# Identification of a Novel Defined Immune-Autophagy-Related Gene Signature Associated With Clinical and Prognostic Features of Kidney Renal Clear Cell Carcinoma

**DOI:** 10.3389/fmolb.2021.790804

**Published:** 2021-12-20

**Authors:** Guangyuan Zhang, Lei Zhang, Si Sun, Ming Chen

**Affiliations:** ^1^ Department of Urology, Zhongda Hospital, Southeast University, Nanjing, China; ^2^ Surgical Research Center, Institute of Urology, Southeast University Medical School, Nanjing, China; ^3^ Department of Urology, Nanjing Lishui District People’s Hospital, Zhongda Hospital Lishui Branch, Southeast University, Nanjing, China

**Keywords:** immune-autophagy, kidney renal clear cell carcinoma, prognosis, biomarkers, autophagy

## Abstract

**Background:** As a common cancer of the urinary system in adults, renal clear cell carcinoma is metastatic in 30% of patients, and 1–2 years after diagnosis, 60% of patients die. At present, the rapid development of tumor immunology and autophagy had brought new directions to the treatment of renal cancer. Therefore, it was extremely urgent to find potential targets and prognostic biomarkers for immunotherapy combined with autophagy.

**Methods:** Through GSE168845, immune-related genes, autophagy-related genes, and immune-autophagy-related differentially expressed genes (IAR-DEGs) were identified. Independent prognostic value of IAR-DEGs was determined by differential expression analysis, prognostic analysis, and univariate and multivariate Cox regression analyses. Then, the lasso Cox regression model was established to evaluate the correlation of IAR-DEGs with the immune score, immune checkpoint, iron death, methylation, and one-class logistic regression (OCLR) score.

**Results:** In this study, it was found that CANX, BID, NAMPT, and BIRC5 were immune-autophagy-related genes with independent prognostic value, and the risk prognostic model based on them was well constructed. Further analysis showed that CANX, BID, NAMPT, and BIRC5 were significantly correlated with the immune score, immune checkpoint, iron death, methylation, and OCLR score. Further experimental results were consistent with the bioinformatics analysis.

**Conclusion:** CANX, BID, NAMPT, and BIRC5 were potential targets and effective prognostic biomarkers for immunotherapy combined with autophagy in kidney renal clear cell carcinoma.

## Instruction

Renal cell carcinoma (RCC), which originates from renal tubular epithelial cells, has always been one of the most common malignant tumors, second only to bladder cancer in adult urinary system malignancies (Siegel et al., 2020). Among them, kidney renal clear cell carcinoma (KIRC) is the most common subtype (accounting for 70–80% of all RCC cases), and it is also one of the most aggressive subtypes with the worst prognosis (Linehan, 2012; Vuong et al., 2019). These tumors are asymptomatic in the early stages of the disease and are usually diagnosed by complications of distant metastasis in the later stages (Hsieh et al., 2017; Tito et al., 2021), 60% of patients with renal clear cell carcinoma die within 1–2 years after diagnosis, and 30% of patients have distant metastases at the time of diagnosis (Casuscelli et al., 2019). Treatment of KIRC can be partial or radical nephrectomy, ablation therapy, and active monitoring of KIRC, while metastatic tumors are treated with therapeutic action, but the overall prognosis is still limited, and immune-related adverse events still need to be improved (Choueiri and Motzer, 2017; Loo et al., 2019; Hofmann et al., 2020; Rizzo et al., 2021). Due to the complex etiology of KIRC and the high heterogeneity of tumor tissues, the treatment and diagnosis of patients are still not ideal. Therefore, it is urgent to find new markers to guide the clinical treatment and diagnosis of KIRC.

Previous studies have confirmed that KIRC is closely related to von Hippel-Lindau (VHL) gene changes (Zhang et al., 2018; Zhang et al., 2020). In addition, ferroptosis-related genes, some miRNAs, and pathways also participate in regulating the process of KIRC regulation (Lu et al., 2021; Zhang et al., 2021). Autophagy plays a vital role in cell physiology, including adaptation to metabolic stress, removal of dangerous substances, renewal during differentiation and development, and prevention of genome damage (Levine and Kroemer, 2008; Levine and Kroemer, 2019). Enormous studies have shown that autophagy is a double-edged sword in the occurrence and treatment of tumors. On the one hand, autophagy can degrade damaged organelles before cell canceration to maintain cell homeostasis and exert a tumor suppressor effect; on the other hand, autophagy can promote the circulation of cell metabolites and meet the nutritional needs of cells. Therefore, in the advanced stage of tumor development, autophagy can provide energy and nutrition for tumor cell proliferation and invasion and can improve tumor cell tolerance to radiotherapy and chemotherapy (Galluzzi et al., 2015; Galluzzi and Green, 2019; Kocaturk et al., 2019).

Since the relationship between iron death and tumors is regulated by many autophagy-related genes, the expression of autophagy-related genes in tumor tissues can be used to assess the prognosis of patients. This study obtained KIRC gene expression information by analyzing The Cancer Genome Atlas (TCGA) database and then analyzed the differential expression of immune autophagy-related genes in the sample, so as to construct a model containing multiple genes to effectively predict the survival of KIRC patients, analyze the risk scoring model correlation with immune status, explore potential mechanisms, provide diagnosis and treatment basis for clinical treatment, and find new therapeutic targets.

## Materials and Methods

### Microarray Data Analysis and Screening of Immune-Autophagy-Related Differentially Expressed Genes

To compare immune-autophagy-related differentially expressed genes (IAR-DEGs) in KIRC, the Gene GEO database was used. The GSE186645 dataset was selected for subsequent analyses. A total of 1,793 human immune-related genes (IRGs) were downloaded from ImmPort database (https://www.immport.org./home), and a total of 223 human autophagy-related genes were downloaded from the Human Autophagy Database (HADb) (http://autophagy.lu/clustering/index.html). The cutoff conditions were set to an adjusted *p*-value <0.05, and the absolute value of log-fold change | log2FC| ≥ 1 was statistically significant for the DEGs. ImageGP was used to create volcano maps and venn maps online.

### Functional Enrichment Analysis of Immune-Autophagy-Related Differentially Expressed Genes in Kidney Renal Clear Cell Carcinoma

Gene Ontology (GO) and Kyoto Encyclopedia of Genes and Genomes (KEGG) pathway analyses were performed by ClusterProfiler software package to explore functional annotation and enrichment pathways, with *p* < 0.05 representing statistically significant differences.

### Survival Analysis and Verification

In order to further evaluate the expression and prognostic value of IAR-DEGs in KIRC, differential analysis and prognostic analysis through “survival” package were conducted. Based on the Cox proportional hazards model and Kaplan–Meier model, the hazard ratio (HR) was calculated, with *p* < 0.05 representing statistically significant differences.

### Construction and Validation of the Immune-Autophagy-Related Differential Expressed Gene-Related Prognostic Model

According to the preliminary screening of IAR-DEGs with differentially expressed and prognostic significance, univariate Cox analysis of overall survival (OS) was performed to identify the survival-related IAR-DEGs with a significant prognosis value (*p* < 0.05). Then, multivariate Cox regression analysis was performed to construct a prediction model based on IAR-DEGs, and the IAR-DEGs were independent prognostic factors. Signatures were established based on the coefficients corresponding to independent prognostic genes. Patients from TCGA-KIRC dataset were divided into low- and high-risk groups weighted by the risk score obtained from the multivariate Cox regression. t-Distributed stochastic neighbor embedding (t-SNE) and principal component analysis (PCA) were used to explore the distribution characteristics of different groups by R packages. Finally, the effectiveness of prognostic indicators was evaluated by the area under the curve (AUC) of “time receiver operating characteristic curve (ROC).”

### Construction of Clinicopathological Correlation Analysis and the Nomogram

Based on “survival” package in R software, combined with the clinicopathological characteristics, the correlation between IAR-DEGs and clinicopathological characteristics was analyzed. Through R package “rms,” the nomogram and calibration curve were obtained. Risk scores associated with prognostic models were used as prognostic factors to evaluate 1-, 3-, and 5-year OS.

### Relationship Between Immune-Autophagy-Related Differentially Expressed Genes and Immune Microenvironment

The relationship between IAR-DEGs expression levels and immune cells was analyzed using the xCell algorithm in the “immunedeconv” R package. The immune score and the effects of gene expression levels on eight immune checkpoint-related genes were also analyzed using the “ggplot2” R package. Finally, TIDE algorithm was used to evaluate two different mechanisms of tumor immune escape using IAR-DEG markers.

### Relationship Between Methylation and Ferroptosis With Immune-Autophagy-Related Differentially Expressed Genes

The third-order RNA sequencing data of genes were obtained based on TCGA dataset, and the association with ferroptosis-related genes and m^6^A-related genes in “ggplot2” R package was analyzed.

### One-Class Logistic Regression Scores of Immune-Autophagy-Related Differentially Expressed Genes in Kidney Renal Clear Cell Carcinoma

Tumor-associated RNA-seq data were obtained from TCGA-KIRC, mRNAsi was calculated by one-class logistic regression (OCLR) algorithm, and the dryness index was obtained.

### Cell Lines, Patient Samples, RNA Extraction, and Quantitative Real-Time PCR

Human kidney cell line HK-2 and human KIRC cell lines, 786-O and caki-1, were originally purchased from the cell repository of Shanghai Institute of Life Sciences. The cells were cultured in 1640 Medium (Gibco, Grand Island, NY, USA), containing 10% fetal bovine serum (FBS) (Gibco), penicillin (25 U/ml), and streptomycin (25 mg/ml), with 5% CO_2_ environment.

In this study, 19 fresh samples, including tumor tissues and adjacent normal kidney tissues, were collected from patients who underwent laparoscopic radical nephrectomy for KIRC from 2019 to 2020 in the Department of Urology, Zhongda Hospital, and stored at 80°C. All patients were diagnosed with KIRC and did not receive any antitumor therapy preoperatively, and none of them had a history of long-term drug use. The clinical characteristics of 19 clear cell RCC (ccRCC) patients are listed in Table 1. The methodology of this study followed the criteria outlined in the Declaration of Helsinki (revised in 2013), and ethical approval was obtained from the Ethics Committee and Institutional Review Board for Clinical Research of Zhongda Hospital (ZDKYSB077). All patients or their relatives who participated were informed and signed an informed consent form.

**TABLE 1:** Clinical characteristics of 19 ccRCC patients.

Sample number	Age	Gender	AJCC	T	N	M	Fuhrman	Tumor size (cm)	Chemotherapy	Radiotherapy
1	59	Female	I	T1	N0	M0	I	3	No	No
2	74	Male	III	T3	N0	M0	II	3.1	No	No
3	52	Male	I	T1	N0	M0	II	6	No	No
4	78	Female	III	T3	N0	M0	III	8.5	No	No
5	82	Female	III	T3	N0	M0	II	4	No	No
6	54	Male	III	T1	N1	M0	I	2.5	No	No
7	46	Male	IV	T3	N0	M1	IV	16	No	No
8	64	Male	I	T1	N0	M0	III	3	No	No
9	23	Male	I	T1	N0	M0	II	2	No	No
10	82	Female	I	T1	N0	M0	III	3.3	No	No
11	77	Male	I	T1	N0	M0	II	3.4	No	No
12	68	Male	I	T1	N0	M0	II	0.8	No	No
13	43	Male	IV	T4	N0	M0	II	9.5	No	No
14	65	Female	III	T3	N1	M0	IV	10	No	No
15	70	Female	II	T2	N0	M0	II	9	No	No
16	58	Male	I	T1	N0	M0	II	4.3	No	No
17	74	Female	I	T1	N0	M0	II	2	No	No
18	40	Male	IV	T3	N0	M1	III	10.7	No	No
19	44	Male	I	T1	N0	M0	I	1.8	No	No

Note. ccRCC, clear cell renal cell carcinoma; AJCC, American Joint Committee on Cancer.

Total RNA was isolated with Total RNA Kit (OMEGAbiotec, Guangzhou, China) according to the manufacturer’s instructions. Complementary DNA was synthesized using the HiScript II Q RT SuperMix (R223-01) reagent kit (Vazyme Biotech Co., ltd., Nanjing, China). The qRT-PCR was performed using the SYBR green PCR mix (vazyme). The specific primers set for mIR-DEGs and GAPDH are listed in Supplementary Table S1. Data were normalized to GAPDH expression levels using the 2^−ΔΔCt^ method.

### Tissue Microarray Construction and Immunohistochemistry

All specimens were fixed in 10% neutral formaldehyde solution and embedded in paraffin. Envision two-step dyeing and DAB color development were used. Primary antibodies BID (ab32060, Abcam, Cambridge, UK), NAMPT (ab236874, Abcam), and BIRC5 (ab76424, Abcam) were used in this study.

### Western Blotting Analysis

Total proteins from HK-2 and human KIRC cells lysed in radioimmunoprecipitation assay (RIPA) (KeyGen, Nanjing, China) buffer were extracted and quantified by bicinchoninic acid (BCA) assay (KeyGen, China). Proteins were analyzed by 10% sodium dodecyl sulfate–polyacrylamide gel electrophoresis (SDS-PAGE), and the gels were transferred onto polyvinylidene fluoride (PVDF) membranes. Then, bovine serum albumin (BSA)-blocked PVDF membranes were incubated with specific primary antibodies BID (1:1,000; ab32060), NAMPT (1:1000; ab236874), BIRC5 (1:5,000; ab76424), and CANX (1:2000; ab133615) overnight at 4°C, followed by incubation of secondary antibodies for 1 h. Finally, bands were visualized using an enhanced chemiluminescence (ECL) kit (vazyme, China).

### Statistical Analysis

The statistical analysis was carried out by R software (version 4.0.2). The Perl programming language (version 5.30.2) was used for data processing. Multivariate Cox regression analyses were used to evaluate prognostic significance. When *p* < 0.05 or log-rank *p* < 0.05, the difference was statistically significant.

## Results

### Identification of Immune-Autophagy-Related Differentially Expressed Genes in Kidney Renal Clear Cell Carcinoma Compared With Normal Renal Tissues

**FIGURE 1:**
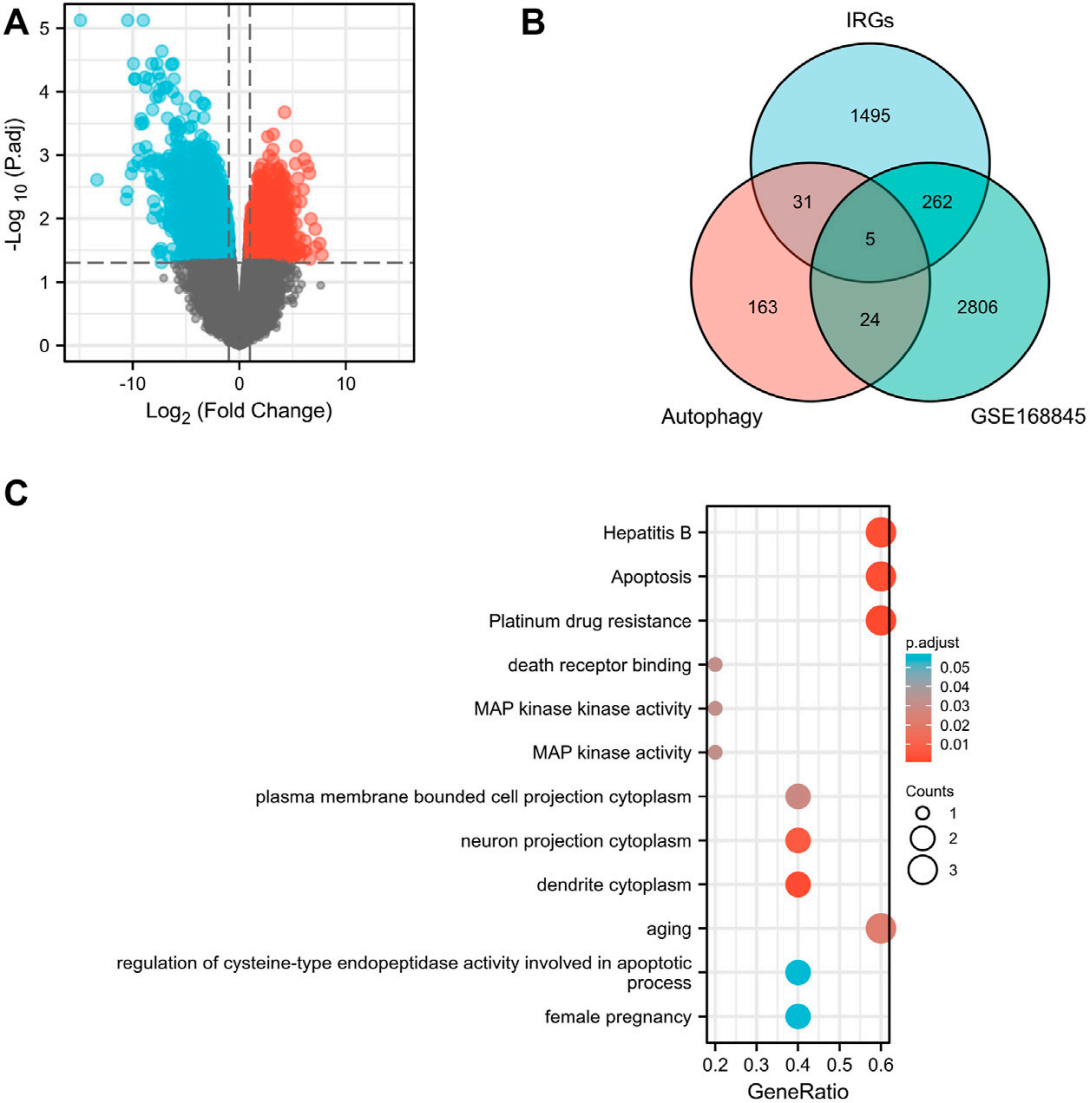
Screening of differentially expressed genes. Volcano plots of differentially expressed genes (DEGs) between normal renal tissues and renal cancer in GSE168845 samples **(A)**. Adjusted *p*-value < 0.05 and log2-fold change (absolute) > 1.3; 635 DEGs were screened with 1,826 upregulated genes and 1,809 downregulated genes. Red represents upregulated genes, and blue indicates downregulated genes. A total of 1,793 human immune-related genes (IRGs) were downloaded from ImmPort database (https://www.immport.org./home), and a total of 223 human autophagy-related genes were downloaded from the Human Autophagy Database (HADb) (http://autophagy.lu/clustering/index.html). Venn diagram showing the five immune-autophagy genes according to the three datasets **(B)**. Graph showing the Gene Ontology (GO) and Kyoto Encyclopedia of Genes and Genomes (KEGG) analysis of the five immune-autophagy genes **(C)**. The five immune-autophagy genes were CANX, MAPK1, BIRC5, NAMPT, and BID.

The volcano map shows 1,826 upregulated DEGs and 1,809 downregulated DEGs that we screened in GSE168845 (Figure 1A). Then, 1,793 human IRGs from ImmPort database and 223 human autophagy-related genes from HADb were analyzed by Venn diagram, and five co-expressed genes were obtained: CANX, MAPK1, BIRC5, NAMPT, and BID (Figure 1B). In the GO/KEGG pathway enrichment analyses, we found five co-expressed differential genes enriched in “aging” in biological process (BP); “dendrite cytoplasm,” “neuron projection cytoplasm,” and “plasma membrane projection cytoplasm” in cellular component (CC); and molecular function (MF) enriched in “MAP kinase activity,” “MAP kinase activity,” and “death receptor binding.” Importantly, the five co-expressed genes in KEGG were mainly enriched in “Platinum drug resistance,” “Apoptosis,” and “Apoptosis-multiple species” (Figure 1C).

### Differential Expression Analysis and Survival Analysis of Immune-Autophagy-Related Differentially Expressed Genes in Kidney Renal Clear Cell Carcinoma

**FIGURE 2:**
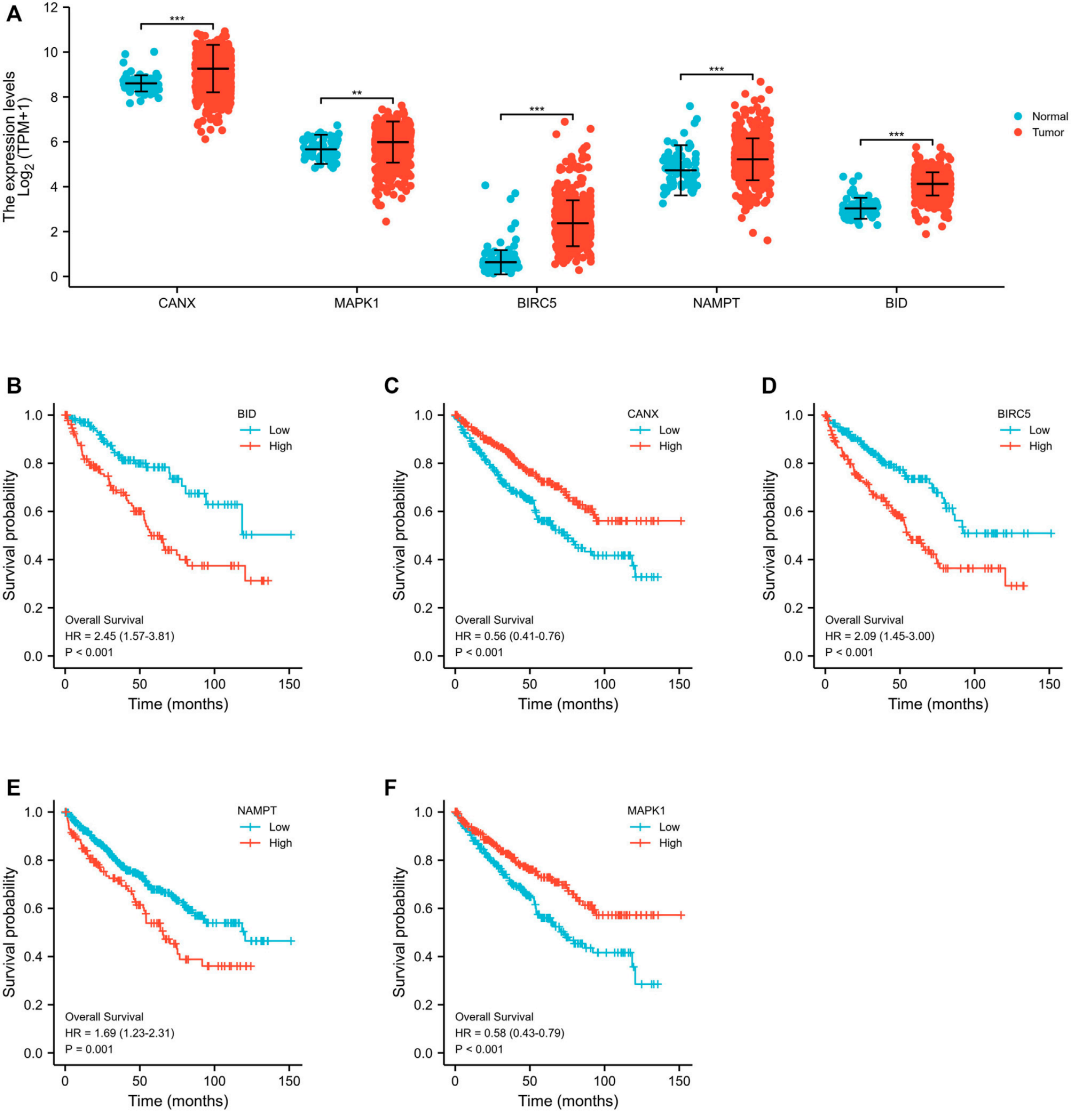
Differential expression and survival analyses of immune-autophagy genes in kidney renal clear cell carcinoma (KIRC). Expression profile of the five immune-autophagy genes in KIRC samples compared with normal tissues **(A)**. Kaplan–Meier plots showing CANX, MAPK1, BIRC5, NAMPT, and BID with prognostic value **(B–F)**.

**FIGURE 3:**
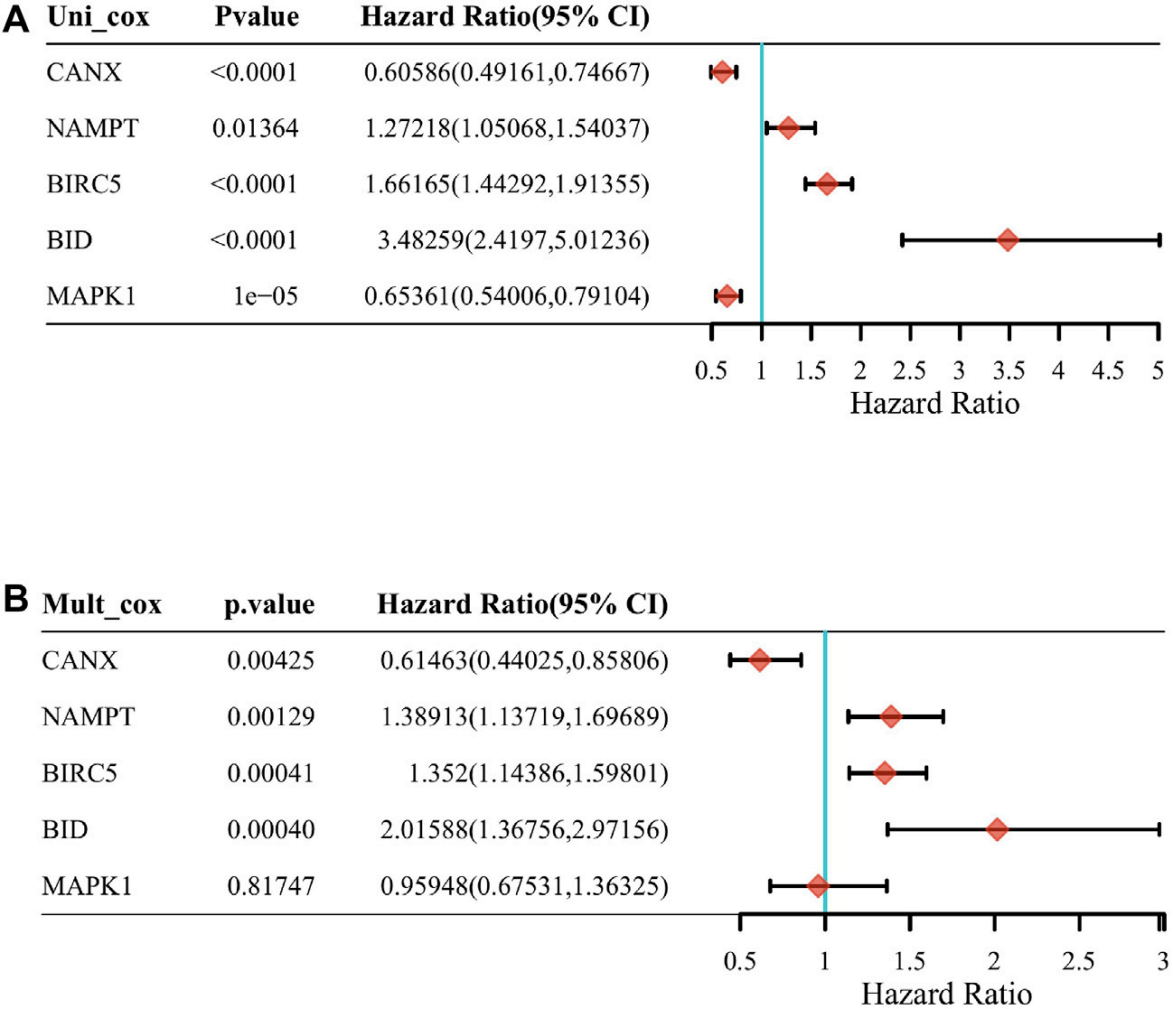
The correlation between the five differentially expressed genes (DEGs) and prognosis. The forest plot shows the results of the univariate Cox regression analyses of the five immune-autophagy genes in The Cancer Genome Atlas–kidney renal clear cell carcinoma (TCGA-KIRC) **(A)**. The forest plot shows the results of the multivariate Cox regression analyses of the five immune-autophagy genes in TCGA-KIRC **(B)**. And CANX, BIRC5, NAMPT, and BID were significant.

Through a screening in TCGA-KIRC database, we compared the expression levels of CANX, MAPK1, BIRC5, NAMPT, and BID in normal kidney tissues and renal clear cell tumor tissues, and we found that their expression levels in tumor tissues were upregulated (Figure 2A). And Kaplan–Meier model analysis shows that the expression levels of the above five DEGs are significantly related to the prognosis, the high expression of CANX and MAPK1 is associated with a good prognosis (Figures 2C,F), and the high expression of BID, BIRC5, and MAPK1 is associated with a poor prognosis (Figures 2B,D,E). Univariate Cox regression analysis (Figure 3A) and multivariate Cox regression analysis (Figure 3B) were used to further explore the correlation between the five DEGs and prognosis, showing that CANX, BIRC5, NAMPT, and BID are independent prognostic factors for KIRC.

### Construction and Validation of the Immune-Autophagy-Related Differentially Expressed Gene Prognostic Risk Model

**FIGURE 4:**
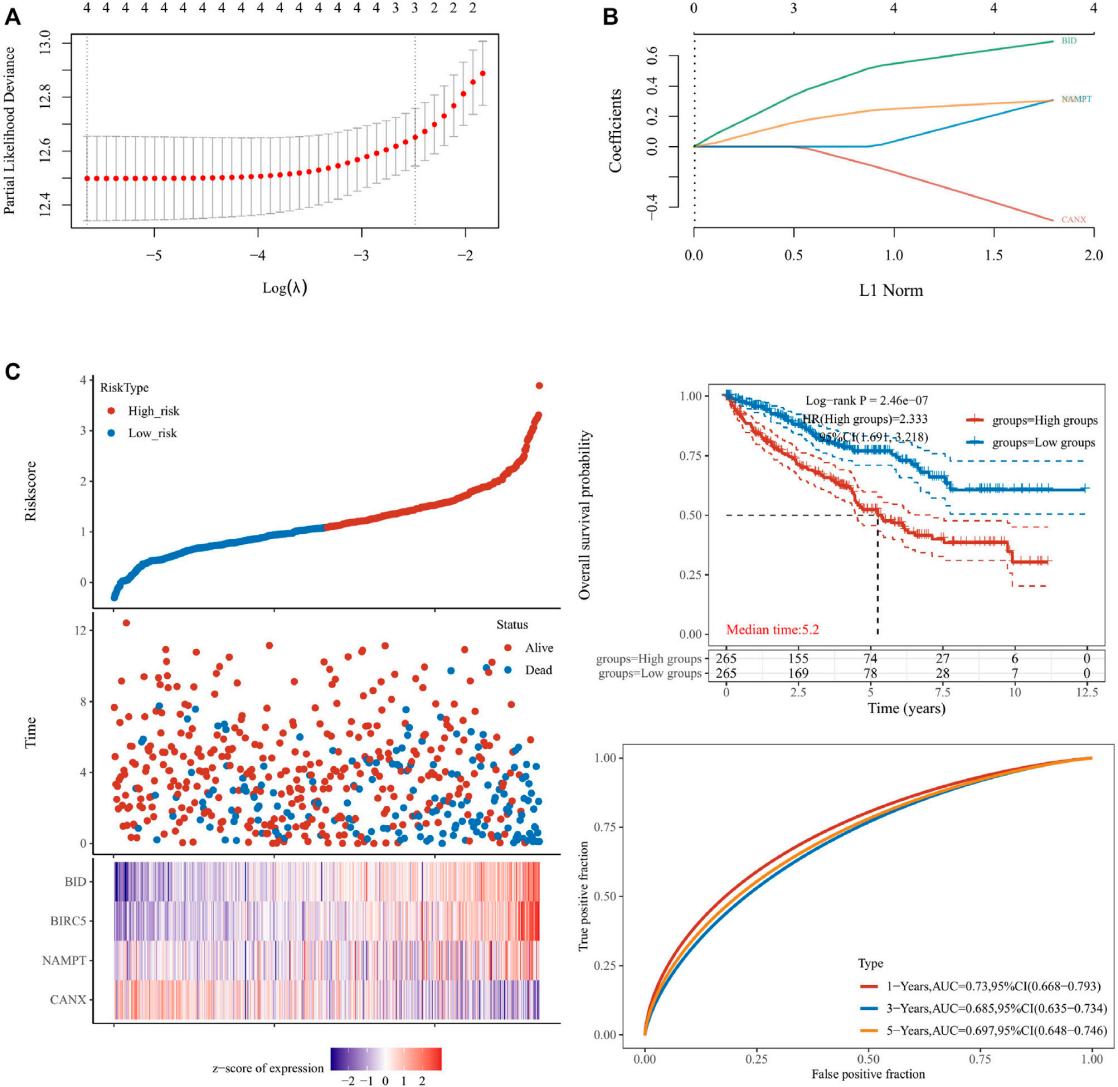
Construction of a prognostic model for the risks associated with differentially expressed genes (DEGs). The calculations for the model according to the multivariate Cox regression analyses **(A,B)**. The prognostic model was analyzed by survival time, survival status, target gene expression heat map, and 1/3/5-year overall survival **(C)**. lambda.min = 0.0035. Riskscore = (−0.4879) * CANX + (0.3075) * NAMPT + (−0.3041) * BIRC5 + (0.694) * BID.

We used lasso Cox regression to construct a prognostic model of DEG-related risks, Risk Score = (−0.4879) * CANX + (0.3075) * NAMPT + (−0.3041) * BIRC5 + (0.694) * BID (Figure 4A, Figure 4B). According to the median risk score (50%), patients were divided into high-risk and low-risk groups. It can be seen in the t-SNE and PCA heat maps that BID, BIRC5, and NAMPT are highly expressed in the high-risk group, and CANX is low in the high-risk group (Figure 4C). If HR = 2.333, the prognosis model can be considered as a risk factor model. The median survival time of the high-risk group was significantly lower than that of the low-risk group (Figure 4C). We used ROC to evaluate the prognostic prediction efficiency of the model, and the results showed that the AUC was 0.73 (1-year OS), 0.685 (3-year OS), and 0.697 (5-year OS) (Figure 4C).

### Relationship Between Immune-Autophagy-Related Differentially Expressed Genes and Clinicopathological Factors and the Construction Nomogram

**FIGURE 5:**
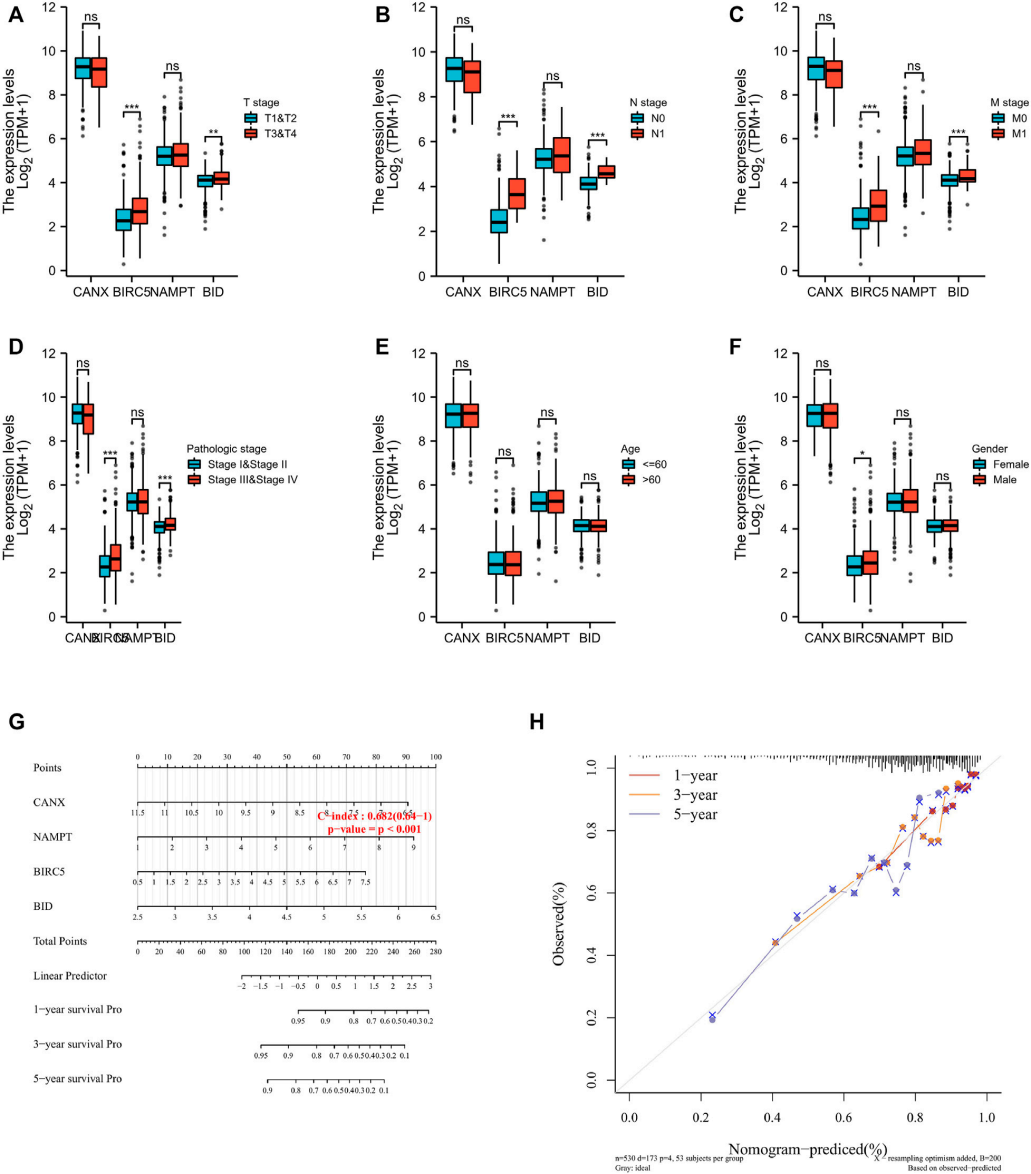
The four immune-autophagy genes significantly correlate with multiple clinicopathological factors in kidney renal clear cell carcinoma (KIRC) patients. The relationships between CANX, BIRC5, NAMPT, and BID and clinicopathological factors in the entire The Cancer Genome Atlas (TCGA) cohort **(A–F)**. Nomogram for predicting 1‐, 3‐, and 5-year overall survival (OS) in the entire TCGA cohort **(G)**. Calibration curves of nomogram on consistency between predicted and observed 1‐, 3‐, and 5-year survival in entire TCGA cohort **(F)**. Dashed line at 45° indicates a perfect prediction.

Regarding the correlation between CANX, BID, NAMPT, BIRC5, and clinicopathological characteristics in the risk prognosis model, our results show that the immune-autophagy-related DEGs associated with T stage, N stage, M stage, and pathological stage are BIRC5 and BID (Figures 5A–D), there is no age-related gene, and the gene related to the patient’s gender is BIRC5 (Figures 5E,F). We used the nomogram to predict 1-, 3-, and 5-year OS in the entire TCGA cohort (Figure 5G). We also found that the 1-, 3-, and 5-year OS on the nomogram is consistent with the calibration curve of predicted probability, and the 1-year OS is the highest (Figure 5H).

### Gene Set Enrichment Analysis of Immune-Autophagy-Related Differentially Expressed Genes

**FIGURE 6:**
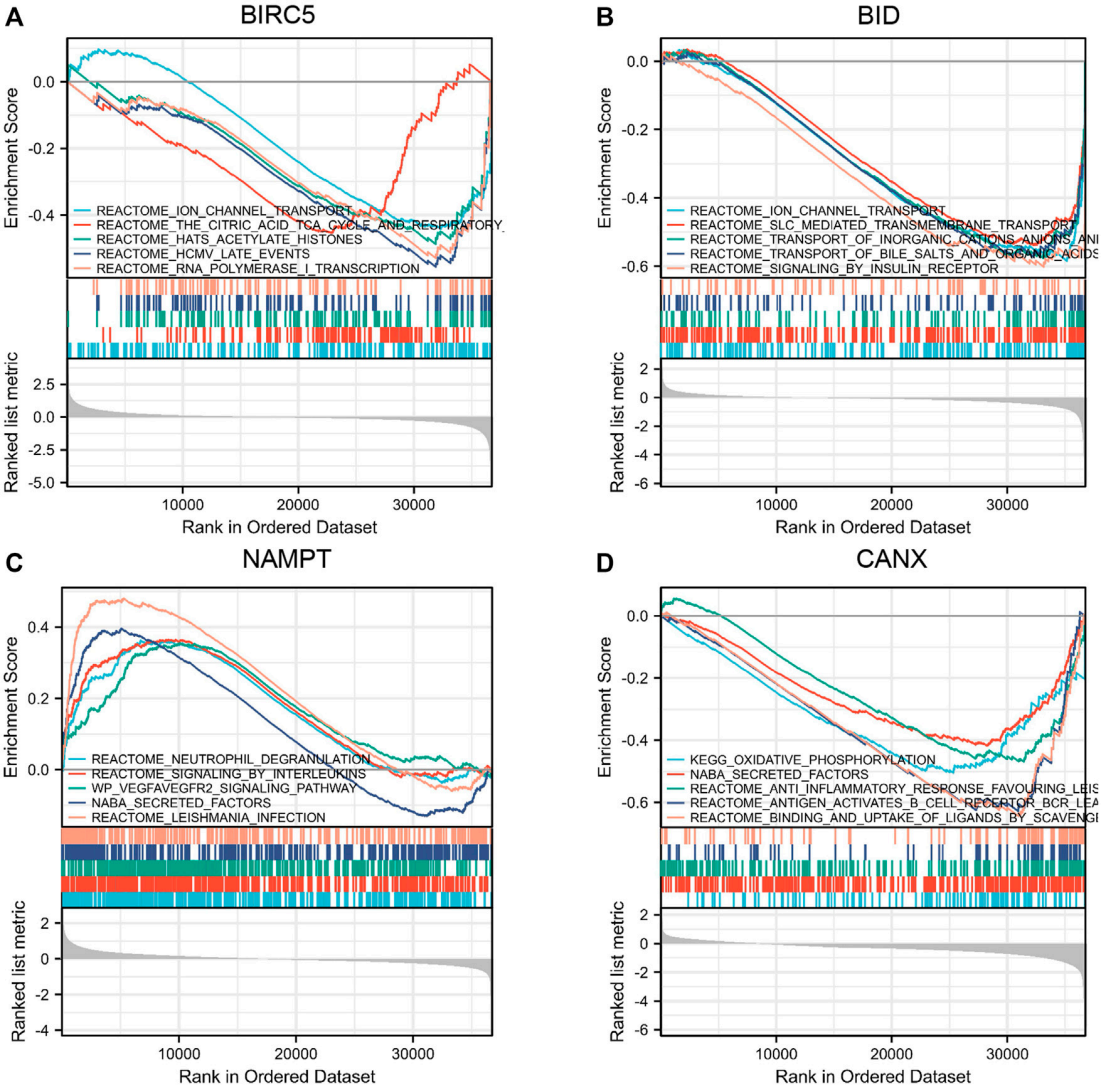
Gene Set Enrichment Analysis (GSEA) of immune-autophagy-related differentially expressed genes (DEGs). Single gene enrichment analysis of BIRC5 **(A)**, BID **(B)**, NAMPT **(C)**, and CANX **(D)**.

We used Gene Set Enrichment Analysis (GSEA) to analyze the KIRC patient data in TCGA-KIRC database. The results showed that both BIRC5 and BID mediate ion channel transport (Figures 6A,B), and both NAMPT and CANX mediate the channel of NABA secretion (Figures 6C,D).

### Correlation Between the Expression of Immune Infiltrating Cells in Kidney Renal Clear Cell Carcinoma Tissues and Immune-Autophagy-Related Differentially Expressed Genes

**FIGURE 7:**
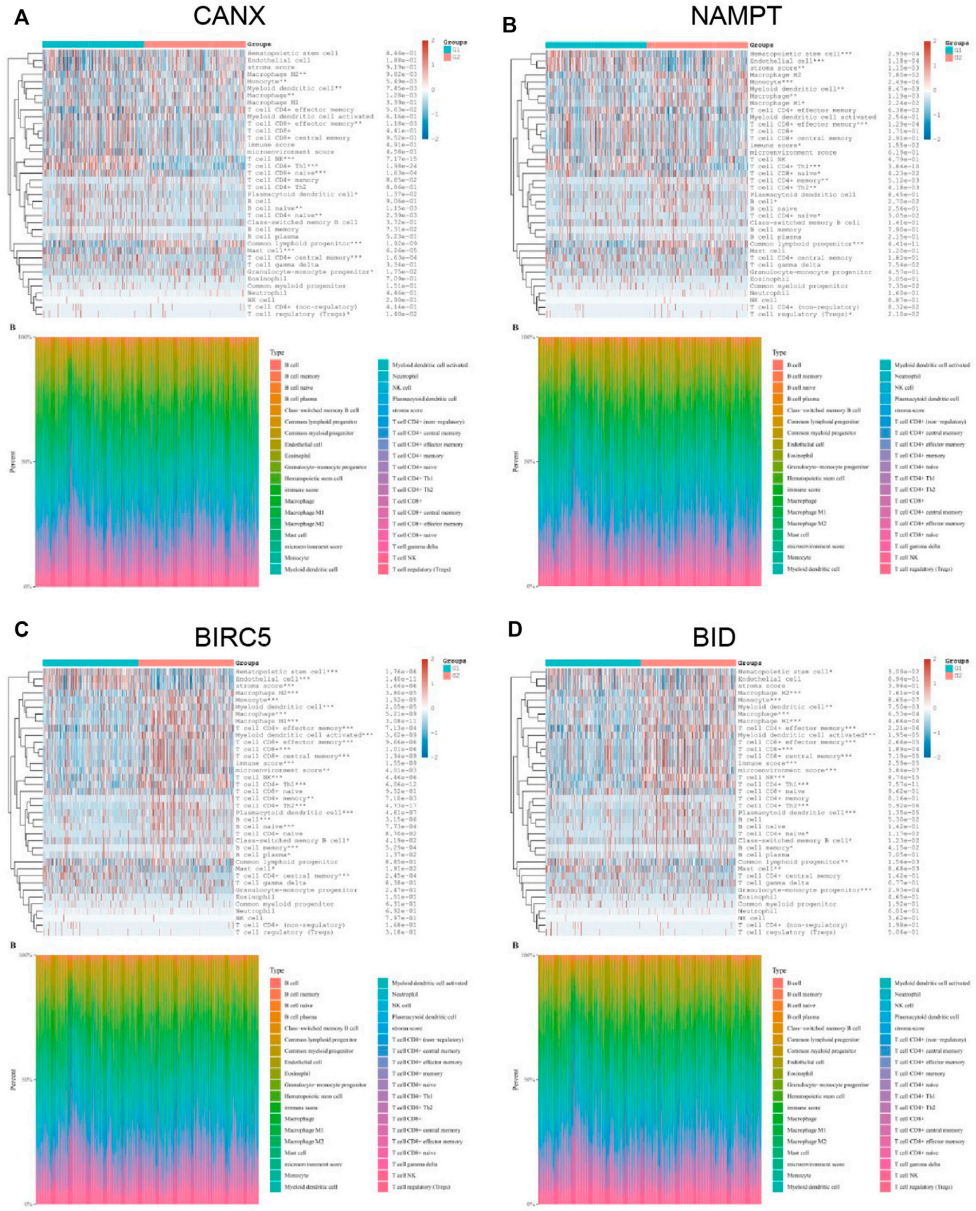
Correlation between the expression of immune infiltrating cells in kidney renal clear cell carcinoma (KIRC) tissues and immune-autophagy-related differentially expressed genes (DEGs). The difference of expression of immune infiltration cells in KIRC tissues with high and low CANX **(A)**, NAMPT **(B)**, BIRC5 **(C)**, and BID **(D)** gene expression. G1 is a low-expression group, and G2 is a high-expression group.

The KIRC population in TCGA-KIRC database was divided into immune-autophagy-related DEG low-expression group (G1) and immune-autophagy-related DEG high-expression group (G2), and the correlation between the expression of immune-infiltrating cells and immune-infiltrating cells was analyzed. The results show that CANX, NAMPT, BIRC5, and BID are highly correlated with the expression levels of a variety of immune infiltrating cells, and the expression levels of monocytes, myeloid dendritic cells, and CD8^+^ effector memory T cells are significantly correlated with CANX, NAMPT, BIRC5, and BID. It suggests that these cells may be related to the progression of KIRC (Figure 7).

### Correlation Between the Expression of Immune Checkpoint in Kidney Renal Clear Cell Carcinoma Tissues and Immune-Autophagy-Related Differentially Expressed Genes

**FIGURE 8:**
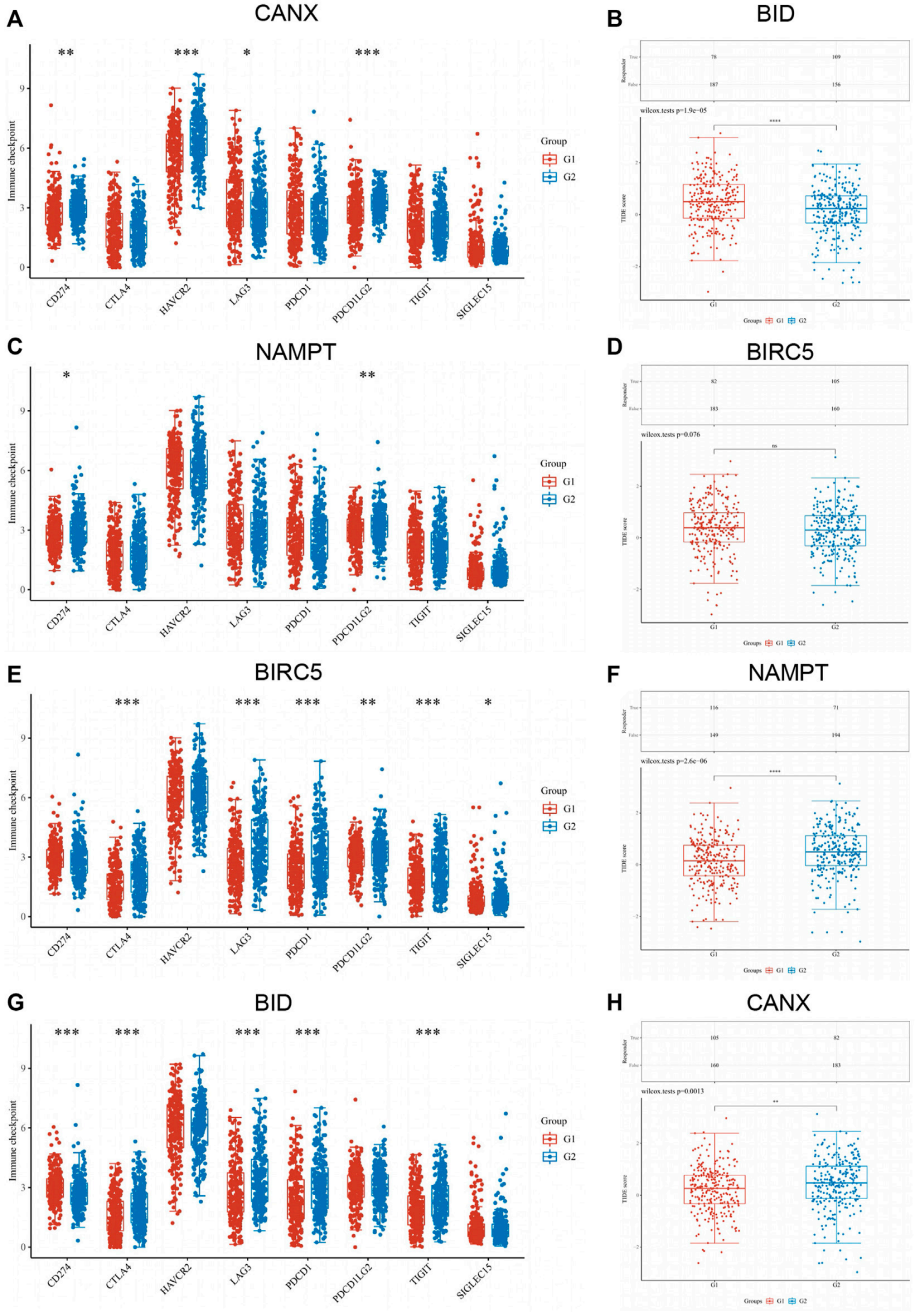
Correlation between the expression of immune checkpoint in kidney renal clear cell carcinoma (KIRC) tissues and immune-autophagy-related differentially expressed genes (DEGs). The difference of expression of immune checkpoint in KIRC tissues with high and low CANX **(A)**, NAMPT **(C)**, BIRC5 **(E)**, and BID **(G)** gene expression. The difference of expression of ICB response in KIRC tissues with high and low BID **(B)**, BIRC5 **(D)**, NAMPT **(F)**, and CANX **(H)** gene expression. G1 is a low-expression group, and G2 is a high-expression group.

Based on the original intention of this study to have a positive effect on the targeted drug therapy of KIRC, we also statistically analyzed the correlation between the expression level of immune checkpoints in KIRC tissues and the expression of immune-autophagy-related DEGs. The results showed that CD274, HAVCR2, LAG3, and PDCDILG2 were significantly correlated with BID (Figure 8A); CD274 and PDCDILG2 were significantly correlated with BIRC5 (Figure 8C); CTLA4, LAG3, PDCD1, PDCDILG2, TIGIT, and SIGLEC15 were significantly correlated with NAMPT (Figure 8E); and CD274, CTLA4, LAG3, PDCD1, and TIGIT were significantly correlated with CANX (Figure 8G). It can be seen that both CD274 and PDCDILG2 have appeared three times. It is speculated that they are sensitive immune checkpoints for KIRC treatment and diagnosis.

In addition, the response of CANX, BID, NAMPT, and BIRC5 with different expression levels to immune checkpoint inhibitors was predicted based on Tumor Immune Dysfunction and Exclusion (TIDE) algorithm (Figures 8B–H). The results indicated that the *p*-values of all immune-autophagy-related genes except NAMPT were <0.05, which indicated that the immune checkpoint inhibitors were effective against KIRC with high expression of CANX, BID, and BIRC5, and the survival period was prolonged after immune checkpoint inhibitor treatment.

### Relationship Between Methylation, Ferroptosis, and Expression of Immune-Autophagy-Related Differentially Expressed Genes

**FIGURE 9:**
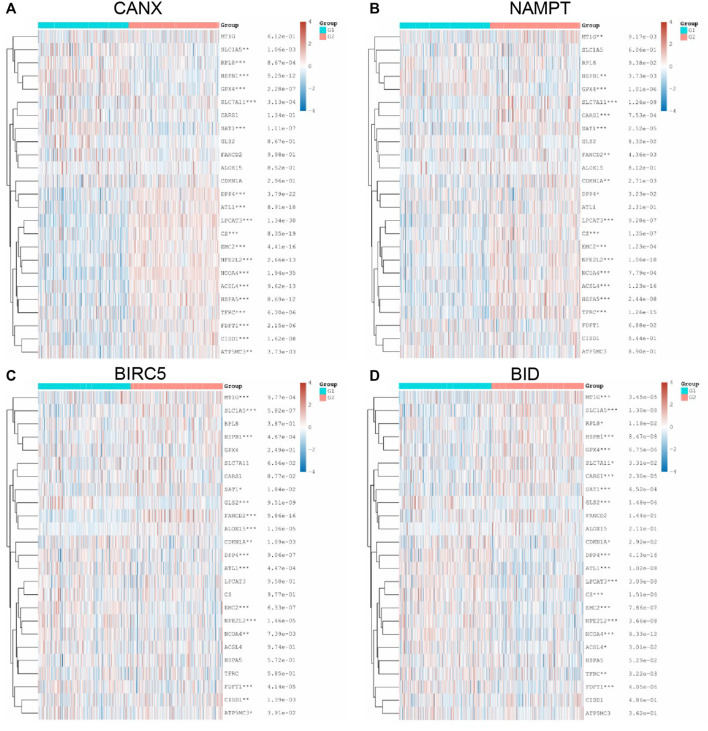
Relationship between methylation, ferroptosis, and expression of immune-autophagy-related differentially expressed genes (DEGs). The difference of expression of ferroptosis-related genes in kidney renal clear cell carcinoma (KIRC) tissues with high and low CANX **(A)**, NAMPT **(B)**, BIRC5 **(C)**, and BID **(D)** gene expression. G1 is a low-expression group, and G2 is a high-expression group.

Following the same analysis method, the results in Figure 9 show that the expression of immune-autophagy-related DEGs is correlated with the expression levels of multiple ferroptosis-related genes, and NCOA4, EMC2, NFE2L2, HSPB1, SAT1, and DPP4 are significantly correlated with CANX, NAMPT, BIRC5, and BID.

**FIGURE 10:**
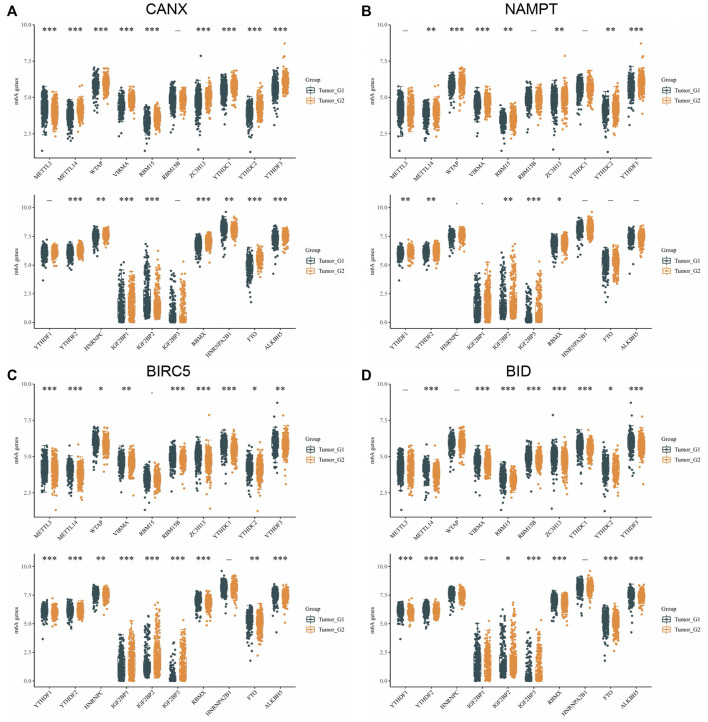
Correlation between m^6^A methylation-related genes and immune-autophagy-related differentially expressed genes (DEGs). The difference of expression of methylation of m^6^A related genes in kidney renal clear cell carcinoma (KIRC) tissues with high and low CANX **(A)**, NAMPT **(B)**, BIRC5 **(C)**, and BID **(D)** gene expression. G1 is a low-expression group, and G2 is a high-expression group.

In addition, we analyzed the correlation between m^6^A methylation-related genes and immune-autophagy-related DEGs by the same method and found that CANX, NAMPT, BIRC5, and BID were significantly correlated with multiple methylated genes (Figure 10). We further verified that m^6^A-related genes were differentially expressed in kidney cancer and normal tissues and were statistically significantly associated with patient prognosis (Supplementary Figures S1, S2). In particular, METTL14, VIRMA, ZC3H13, TYHDC2, YTHDF3, YTFDF2, IGF2BP2, and RBMX were significantly associated with four immune-autophagy-related DEGs.

### Assessment of the One-Class Logistic Regression Scores of Immune-Autophagy-Related Differentially Expressed Genes in Kidney Renal Clear Cell Carcinoma

**FIGURE 11:**
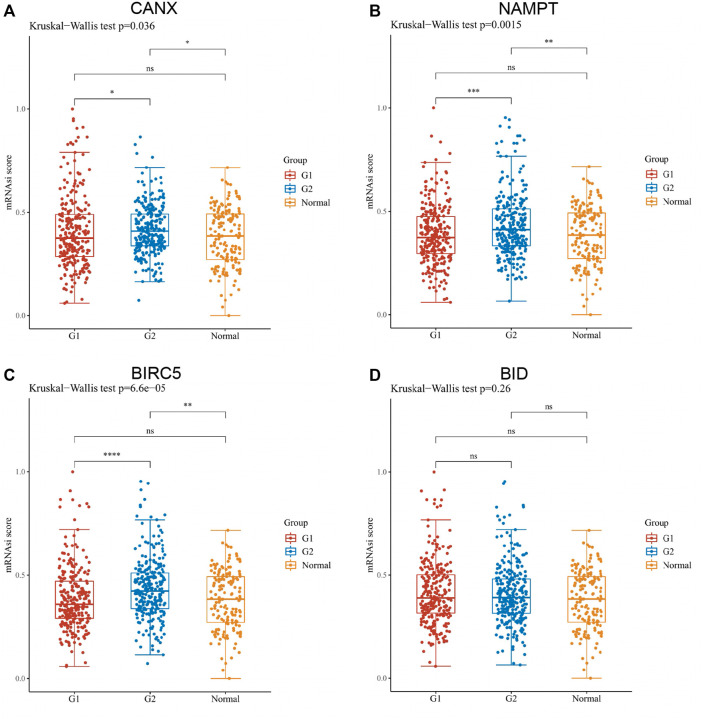
Assessment of the one-class logistic regression (OCLR) scores of immune-autophagy-related differentially expressed genes (DEGs) in kidney renal clear cell carcinoma (KIRC). Scatter diagram illustrating the relationship between CANX **(A)**, NAMPT **(B)**, BIRC5 **(C)**, and BID **(D)** and OCLR score in KIRC. The horizontal axis in the figure represents the gene expression distribution, and the vertical axis is the OCLR score distribution. G1 is a low-expression group, and G2 is a high-expression group.

By OCLR scores, we found that, except for BID, the expression levels of CANX, NAMPT, and BIRC5 were significantly different from the dryness degree of KIRC (Figure 11). These results suggested that CANX, NAMPT, and BIRC5 may influence the degree of similarity between KIRC cells and stem cells and thus affect the BP and degree of dedifferentiation of tumors.

### Validation of the Expression of Differentially Expressed Genes in Clinical Tissue Samples

**FIGURE 12:**
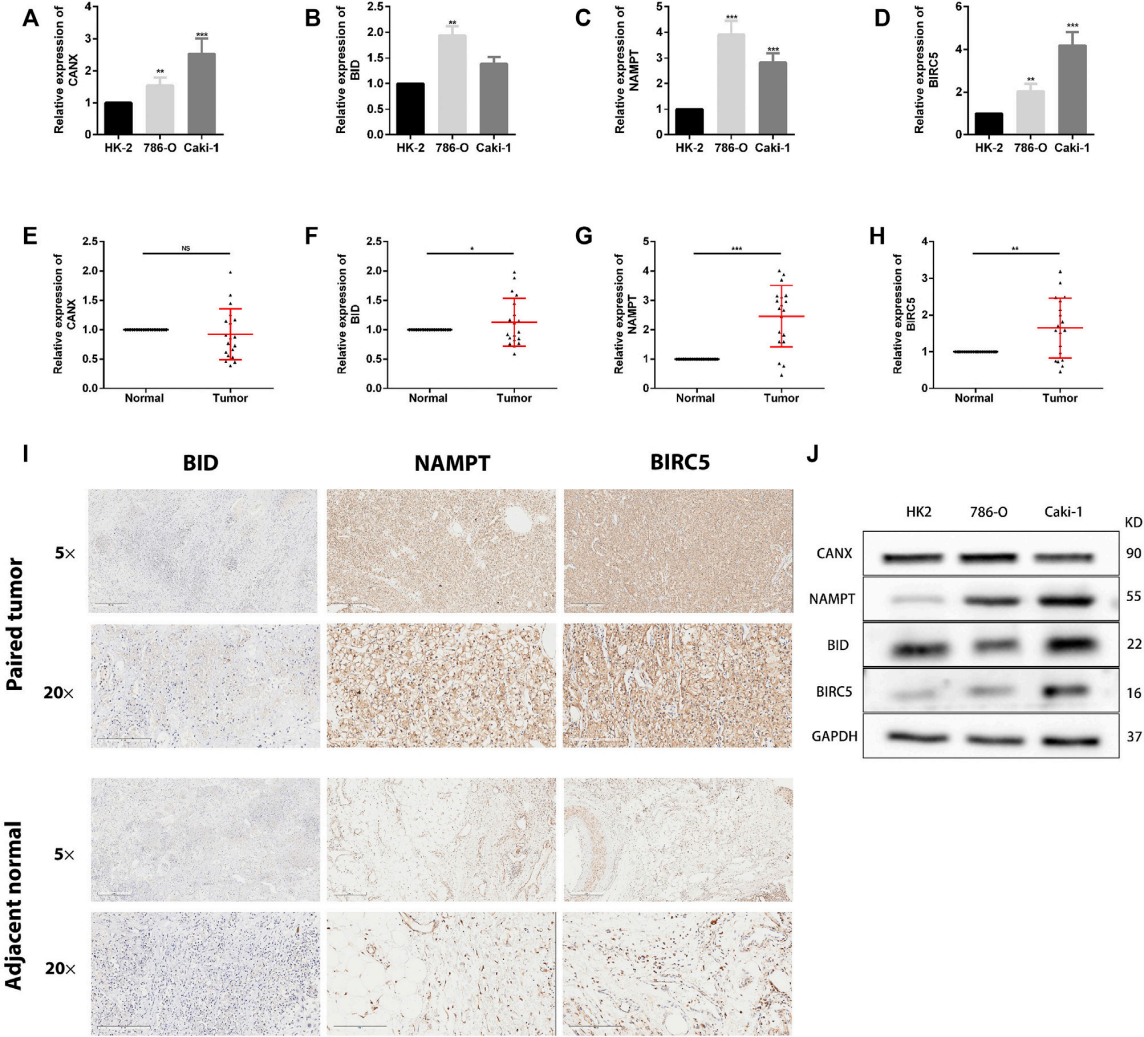
The expression of these genes in human kidney renal clear cell carcinoma (KIRC) specimens, adjacent normal tissues, and cell lines. **(A–D)** qRT-PCR analysis of CANX **(A)**, BID **(B)**, NAMPT **(C)**, and BIRC5 **(D)** in KIRC cell lines. GAPDH was used as a loading control. **(E–H)** qRT-PCR analysis of CANX **(E)**, BID **(F)**, NAMPT **(G)**, and BIRC5 **(H)** in paired KIRC tissues (*n* = 19). **(I)** Representative images of BID, NAMPT, and BIRC5 protein immunochemistry in KIRC tissues compared with adjacent normal kidney tissues. Magnification, ×5 and ×20. **(J)** Western blotting analysis of related differentially expressed genes (DEGs) expression levels in normal kidney cell line (HK-2 cells) and two KIRC cell lines (786-O and caki-1). *p < 0.05, **p < 0.01, and ***p < 0.001.

To detect the expression of four genes (CANX, BID, NAMPT, and BIRC5) in KIRC, we performed the qRT-PCR in KIRC cells and clinical tissue samples. We verified the expression levels of four genes in normal kidney cell lines (HK-2 cells) and two KIRC cell lines (786-O and caki-1). The results showed that the expression levels of four genes were significantly increased in KIRC cells compared with normal kidney cells (Figures 12A–D). In addition, Western blotting results showed that protein levels of NAMPT and BIRC5 were expressed at increased levels in RCC cell lines 786 and caki-1, but there was no significant difference in protein levels of BID and CANX (Figure 12J). BID, NAMPT, and BIRC5 were detected with the same results in tumor tissues and with adjacent normal kidney tissues, while CANX was not significantly different (Figures 12E–H). Then we detected the protein expression of BID, NAMPT, and BIRC5 in the tissues by immunohistochemistry (IHC). Results demonstrated that NAMPT and BIRC5 were significantly increased in KIRC tissues compared with adjacent normal kidney tissues. However, BID was negative in most tissues (Figure 12I).

## Discussion

Early symptoms of clear cell RCC are insidious, and patients often have metastases at the time of diagnosis. Because of its complex biological characteristics, surgical resection is not easy, more than one-tenth of patients will have a fatal relapse within 5 years after traditional partial or radical nephrectomy, and it is not sensitive to radiotherapy and chemotherapy (Fu et al., 2016; Pandey et al., 2020). In recent years, targeted therapies against vascular endothelial growth factor (VEGF) and immunotherapy have gradually replaced nonspecific immune methods as the primary medical treatment for patients with KIRC (Şenbabaoğlu et al., 2016; Barata and Rini, 2017; Smith et al., 2018; Dizman et al., 2020). Even though researchers have made some progress in this area, the selection of biomarkers, the combined use of drugs, and the ambiguity of immune checkpoints are still crucial issues that cannot be ignored (Tang et al., 2013; Ghatalia et al., 2017; Mao et al., 2021). Therefore, studying the mechanism of the occurrence and development of clear cell RCC has become a clinically urgent need to solve the problem. We understand that autophagy-related genes are closely related to cancer, and their expression levels differ at different cancer stages. Few studies are linking the prognosis and treatment of KIRC with autophagy-related genes. We hope to illustrate this kind of relevance through some analyses.

In this study, we first conducted Venn diagram analysis from the genes in the GSE168845, ImmPort database, and HADb to obtain five co-expressed immune-autophagy-related DEGs, and we discarded MAPK1 after performing multivariate Cox regression analysis. We found that the expression levels of CANX, BIRC5, BID, and NAMPT in tumor tissues were significantly higher than their expression levels in normal tissues, indicating that they are all significantly related to tumor occurrence and development. The Kaplan–Meier model we established shows that patients with high expression of BID and BIRC5 have a worse prognosis. By contrast, patients with high expression of CANX have a better prognosis, which is consistent with the results of our DEG-related risk prognosis model constructed by lasso Cox regression. In order to better understand the correlation between these four immune-autophagy-related DEGs and tumors, we also statistically analyzed their correlation with tumor stage, histopathological morphology, patient age, patient gender, and other clinicopathological characteristics. In addition, the calibration curves and nomogram showed a good prediction effect. The expression level of immune-autophagy-related DEGs is also significantly correlated with immune infiltration, immune checkpoints, methylation, and iron death. Among these results, the performance of BIRC5 and BID is particularly outstanding; immune infiltrating cells such as monocytes, myeloid dendritic cells, CD8^+^ effector memory T cells, and immune checkpoint CD274 deserve special attention. Furthermore, we performed the qRT-PCR analysis and IHC in clinical samples and found that the expression of NAMPT and BIRC5 was significantly higher in ccRCC tissues when compared with that in adjacent normal tissues. More *in vivo* and *in vitro* experiments are needed to authenticate these findings.

Baculoviral IAP repeat containing 5 (BIRC5) has been broadly studied among cancer therapeutic targets, and its main function is to suppress cell death (Li et al., 2019). Numerous researches have shown that BIRC5 contributes to tumor cell immune escape by inhibiting apoptosis and confirmed that its expression is strongly correlated with prognostic status and OS in various cancers (e.g., lung, colorectal, prostate, and ovarian cancers) (Cao et al., 2019; Filipchiuk et al., 2020; Wang et al., 2021). However, there are no relevant studies to explore the therapeutic effects of BIRC5 small-molecule inhibitors in tumors (Li et al., 2019). BH3-Interacting Domain Death Agonist (BID), as the activator and integrator, is involved in apoptosis-related pathways (Billen et al., 2008; Gryko et al., 2014). Lee found that BID proteins are involved in mediating DNA damage responses and promoting normal cell apoptosis (Lee et al., 2004). Regrettably, there are no relevant studies that explored the specific action mechanism and related functions of BID in tumors. Nonetheless, studies have suggested that TAT-BID + DOX may be a potentially effective combination for the treatment of cancers, but no final conclusions can be drawn due to the absence of protein and cytokine pathways (Zhang et al., 2004; Goncharenko-Khaider et al., 2010; Orzechowska et al., 2015). Additionally, nicotinamide phosphoribosyltransferase (NAMPT) is an important cofactor involved in various biochemical reactions (Travelli et al., 2018). It is now generally believed that NAMPT is highly expressed in cells with active proliferation, especially tumor cells (Garten et al., 2015), which implicates NAMPT-targeted small-molecule inhibitors as potential tumor therapeutic agents. Existing related studies have identified NAMPT inhibitors and their vectors as important directions for anticancer therapy (Garten et al., 2009; Bi and Che, 2010; Lucena-Cacace et al., 2018; Zhang et al., 2019; Galli et al., 2020). Ultimately, calnexin (CANX), an endoplasmic reticulum lectin chaperone protein (Ellgaard et al., 2016; Kozlov and Gehring, 2020), has been confirmed to be upregulated in tumors including lung cancer and oral squamous carcinomas, and its ability to inhibit the proliferation of CD4^+^ T and CD8^+^ T cells in tumor tissues (Kobayashi et al., 2015; Alam et al., 2019; Chen et al., 2019), as well as the release of cytokines (PD-1, IFN-γ, and TNF), which eventually promotes tumor growth. Unfortunately, there is no clear mechanism for the regulation of CANX in tumors (Li et al., 2001; Kobayashi et al., 2015; Ellgaard et al., 2016; Alam et al., 2019; Chen et al., 2019; Kozlov and Gehring, 2020).

In summary, BIRC5, BID, NAMPT, and CANX, which were finally screened by bioinformatics analysis of autophagy-immune-related genes, are important in tumorigenesis, progression, and apoptosis. Regrettably, there are no relevant studies to explore their specific mechanisms and functions in KIRC and the potential efficacy of relevant targeted small-molecule inhibitors. We believe that this will be an important concept and direction for the academic community to investigate the mechanism and function of autophagy-immunity in renal cancer afterward.

Our study still had some limitations. The dataset we used to construct and validate the IAR-DEG prognostic signature was obtained from ImmPort database. We failed to locate suitable data from other immunological databases to verify the reliability of the screened genes. We only performed preliminary expression studies on these four IAR-DEGs in the signature. However, further functional analysis and mechanistic studies were not carried out.

## Conclusion

In this study, we obtained immune-autophagy-related genes with independent prognostic value through comprehensive bioinformatics analysis. We established the prognostics risk model. A significant correlation was found among immune-autophagy-related genes and the immune score, immune checkpoint, methylation, ferroptosis, and OCLR score. As a result, CANX, BID, NAMPT, and BIRC5 were potential targets and effective prognostic biomarkers for immunotherapy combined with autophagy.

## Data Availability

The original contributions presented in the study are included in the article/Supplementary Material. Further inquiries can be directed to the corresponding authors.
