# Supplementary material for: Identification of a Novel Defined Immune-Autophagy-Related Gene Signature Associated With Clinical and Prognostic Features of Kidney Renal Clear Cell Carcinoma
Source: Front Mol Biosci. 2021 Dec 20;8:790804. doi: 10.3389/fmolb.2021.790804 (PMC8721006; doi:10.3389/fmolb.2021.790804)
Supplement: Supplementary file 1 [file Table1.DOCX]

**Table S1:** The primer sequences used in the present study.

| Gene | Forward primer (5′-3′) | Reverse primer (5′-3′) |
| --- | --- | --- |
| CANX | CCAAGGTTACTTACAAAGCTCCA | GGCCCGAGACATCAACACA |
| BID | ATGGACCGTAGCATCCCTCC | GTAGGTGCGTAGGTTCTGGT |
| NAMPT | CGGCAGAAGCCGAGTTCAA | GCTTGTGTTGGGTGGATATTGTT |
| BIRC5 | AGGACCACCGCATCTCTACAT | AAGTCTGGCTCGTTCTCAGTG |
| GAPDH | ACCATCTTCCAGGAGCGAGAT | GGGCAGAGATGATGACCCTTT |
