# Supplementary figures and images for: Identification of a Novel Defined Immune-Autophagy-Related Gene Signature Associated With Clinical and Prognostic Features of Kidney Renal Clear Cell Carcinoma
Source: Front Mol Biosci. 2021 Dec 20;8:790804. doi: 10.3389/fmolb.2021.790804 (PMC8721006; doi:10.3389/fmolb.2021.790804)

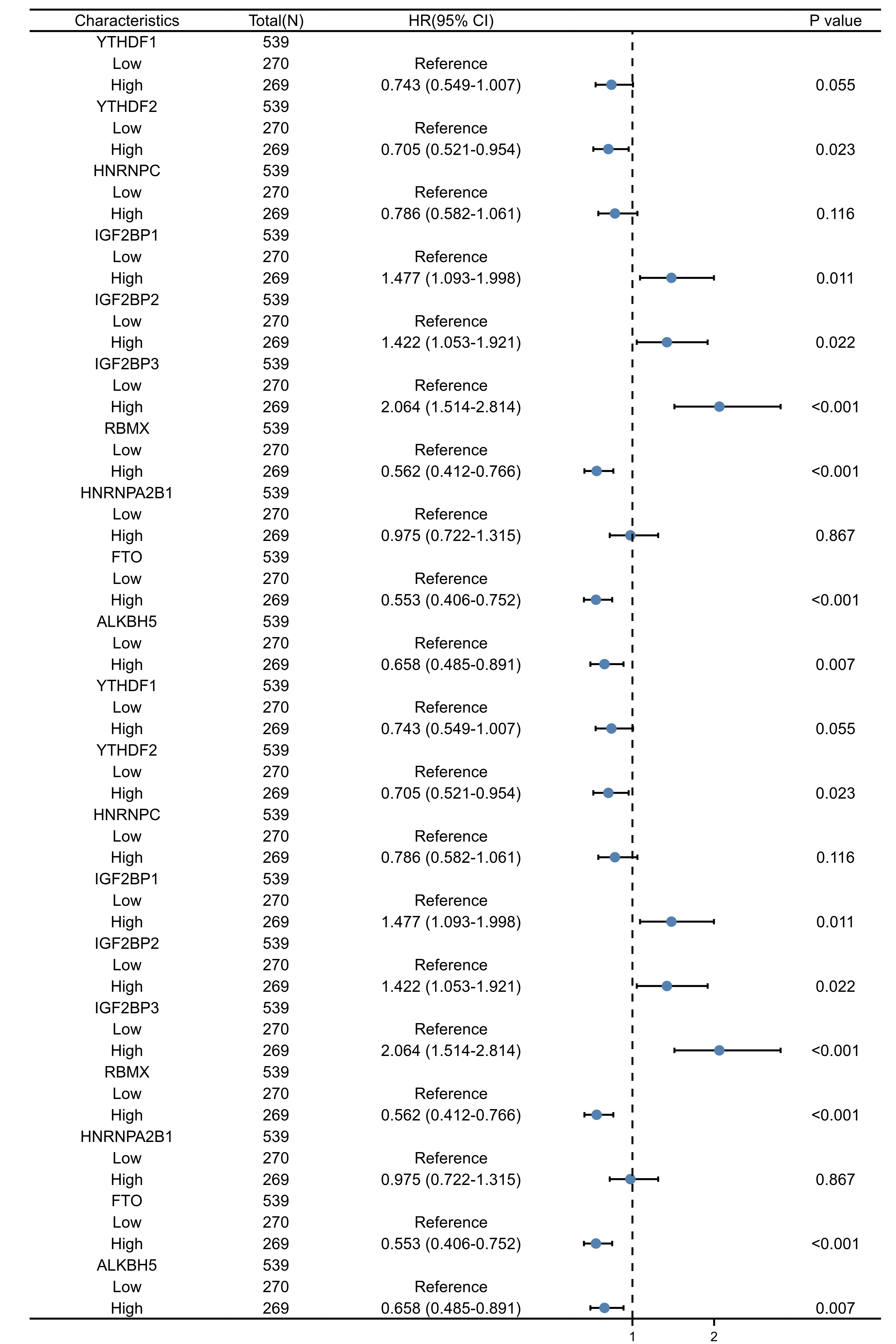

Supplement: Supplementary file 2 [file Image1.JPEG]

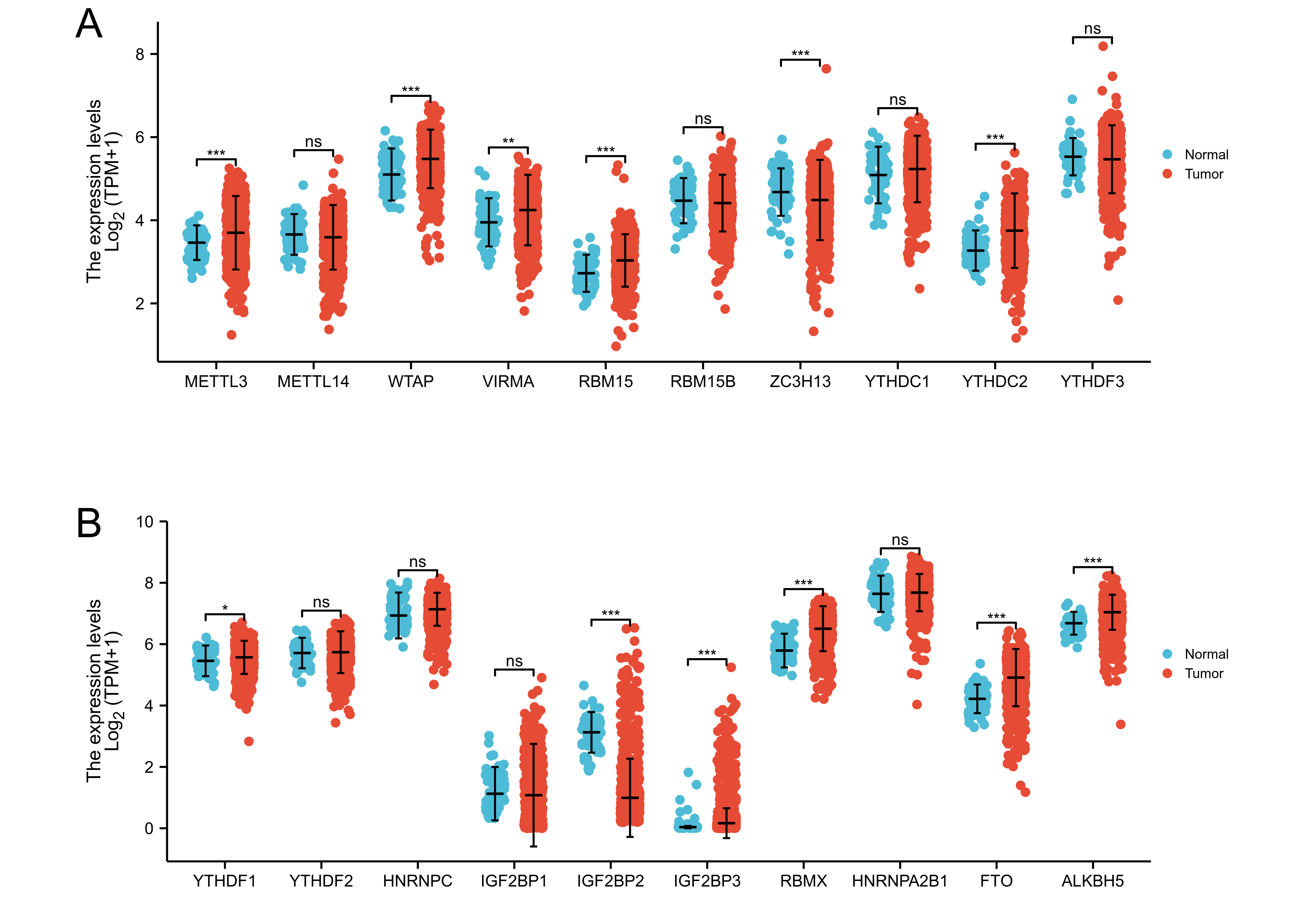

Supplement: Supplementary file 3 [file Image2.JPEG]
